# Trends in gastrointestinal cancer incidence in Iran, 2001-2010: a joinpoint analysis

**DOI:** 10.4178/epih.e2016056

**Published:** 2016-12-05

**Authors:** Mehdi Darabi, Mohsen Asadi Lari, Seyed Abbas Motevalian, Ali Motlagh, Shahram Arsang-Jang, Maryam Karimi Jaberi

**Affiliations:** 1 Department of Epidemiology, School of Public Health, Iran University of Medical Sciences, Tehran, Iran; 2 Oncopathology Research Center, Iran University of Medical Sciences, Tehran, Iran; 3 Department of Radiotherapy, Shahid Baheshti University of Medical Sciences, Tehran Iran; 4 Department of Epidemiology and Biostatistics, Faculty of Health, Qom University of Medical Sciences, Qom, Iran

**Keywords:** Incidence, Gastrointestinal neoplasms, Trends, Joinpoint, Iran

## Abstract

**OBJECTIVES:**

The main purpose of this study was to evaluate changes in the time trends of stomach, colorectal, and esophageal cancer during the past decade in Iran.

**METHODS:**

Cancer incidence data for the years 2001 to 2010 were obtained from the cancer registration of the Ministry of Health. All incidence rates were directly age-standardized to the world standard population. In order to identified significant changes in time trends, we performed a joinpoint analysis. The annual percent change (APC) for each segment of the trends was then calculated.

**RESULTS:**

The incidence of stomach cancer increased from 4.18 and 2.41 per 100,000 population in men and women, respectively, in 2001 to 17.06 (APC, 16.7%) and 8.85 (APC, 16.2%) per 100,000 population in 2010 for men and women, respectively. The corresponding values for colorectal cancer were 2.12 and 2.00 per 100,000 population for men and women, respectively, in 2001 and 11.28 (APC, 20.0%) and 10.33 (APC, 20.0%) per 100,000 in 2010. For esophageal cancer, the corresponding increase was from 3.25 and 2.10 per 100,000 population in 2001 to 5.57 (APC, 12.0%) and 5.62 (APC, 11.2%) per 100,000 population among men and women, respectively. The incidence increased most rapidly for stomach cancer in men and women aged 80 years and older (APC, 23.7% for men; APC, 18.6% for women), for colorectal cancer in men aged 60 to 69 years (APC, 24.2%) and in women aged 50 to 59 years (APC, 25.1%), and for esophageal cancer in men and women aged 80 years and older (APC, 17.5% for men; APC,15.3% for women) over the period of the study.

**CONCLUSIONS:**

The incidence of gastrointestinal cancer significantly increased during the past decade. Therefore, monitoring the trends of cancer incidence can assist efforts for cancer prevention and control.

## INTRODUCTION

Cancer is an important public health challenge in Iran, as well as in most countries worldwide [1]. In Iran, after cardiovascular diseases and accidents, cancer is the third most common cause of death. Although the rate of cancer incidence in developed countries is generally two times greater than that in developing countries, the number of people affected by cancer yearly in developing countries is higher, and their disease is also more deadly [2]. Cancer in Iran shows considerably different patterns than those that exist in developed countries. For example, stomach and esophageal cancers, which are the most prevalent types of cancer in Iran, are much less common in some other countries, especially the US, where they are not among the top 10 prevalent types of cancer [3]. In this study, we used cancer registration data. Cancer registration is a tool for measuring cancer incidence in a defined population during a specific period of time in order to evaluate and control for the effects of cancer in society [4]. The obtained data can be used for studying trends in cancer incidence, trends, patient follow-up, and survival [5]. Moreover, cancer registration is an important part of cancer control programs, and registration data can be used widely in etiologic research, evaluation of the effects of preventive measures, and program development in healthcare services [6]. Stomach, colorectal, and esophageal cancers have a high incidence among Iranian men and women; colorectal and stomach cancers are among the five most prevalent cancers, and esophageal cancer is among the 10 most prevalent cancers.

Iran is experiencing changes in age demographics, economic development, and social transformations, which can have significant effects on cancer patterns and changes in cancer trends. The main purpose of the current study was to evaluate the time trends of age-standardized incidence rates (ASRs) and identify changes in the incidence of gastrointestinal cancers (stomach, colorectal, and esophageal) from 2001 to 2010.

## MATERIALS AND METHODS

The cancer incidence statistics for 2001 to 2010 were obtained from the National Cancer Control Program in Iran (Ministry of Health). The Iran cancer registry program is designed to register all cancer cases in the country. All pathology centers and other information sources such as hospitals’ medical records, mortality data from provincial governments, hematology-oncology centers, radiotherapy centers, and so on are obliged to report their data to the Cancer Office of Disease Control and Prevention (CDC). The CDC then annually enters the data in a computer program, identifies duplicates and reconciling the new data with the data from previous years. The executive arms of the program include all medical universities in the country. The three cancer sites included in this study were defined according to the International Classification of Diseases, 10th revision. Stomach cancer was defined by code C16, esophageal cancer by code C15, and colorectal cancer by codes C18 to C21. We used ASRs in order to compare populations with different age structures, with weights obtained from the world standard population [7]. We then compared the incidence rates for five specific age groups (40-49 years, 50-59 years, 60-69 years, 70-79 years, and 80 years and older).

For analysis, we used joinpoint regression analysis to identify the years when significant changes took place in the ASRs. Joinpoint regression analysis fits a series of joined straight lines to the ASRs on a log scale [8]. Straight line segments are joined at joinpoints, where the incidence trend changes its slope to a statistically significant extent. We allowed a maximum of two joinpoints, and an overall significance level of 5% was used for the comparisons of models applied to each data series. The best-fitting model was estimated separately for men and women. The Joinpoint version 4.2.0.2 (https://surveillance.cancer.gov/joinpoint/) was used for the statistical analysis.

## RESULTS

### Stomach cancer

**Figure 1.**
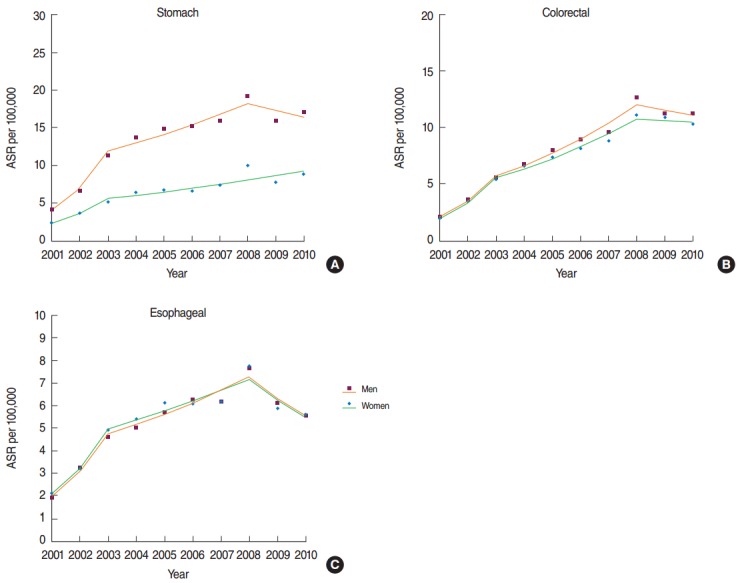
Trends in the age-standardized incidence rate (ASR) for gastrointestinal cancers (A: stomach, B: colorectal, and C: esophageal) in Iran, 2001-2010.

**Figure 2.**
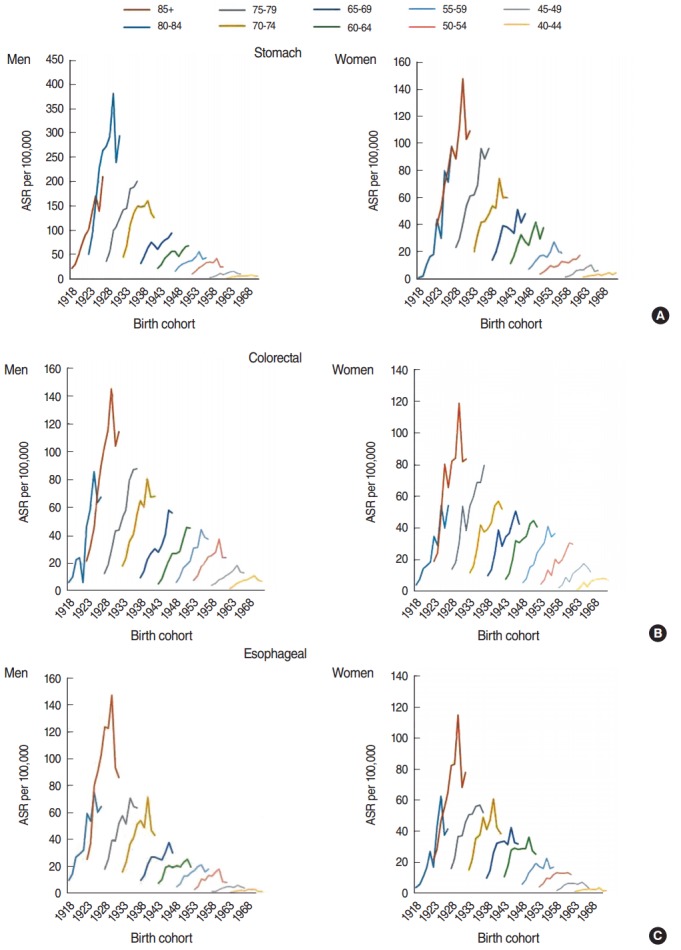
Trends in the age-standardized incidence rate (ASR) of gastrointestinal cancers (A:stomach, B: colorectal, and C: esophageal) by age group.

For stomach cancer, a significant increase in incidence was observed for both men and women, with annual percent changes (APCs) of 16.7% (95% confidence interval [CI], 14.6 to 18.8%) and 16.2% (95% CI, 7.7 to 25.4%), respectively, from 2001 to 2010. The ASR for men was higher than for women (Table 1). In men, the incidence significantly increased from 2001 to 2008; however, this trend changed in 2008, after which no significant increase was observed. However, in women, the incidence increased throughout the study period (Figure 1). In men and women, the highest incidence changes were observed in the age group of 80 years old above and higher (APC, 23.7% for men; APC, 18.6% for women) (Table 2). The ASRs of stomach cancer by birth cohort is plotted in Figure 2. The incidence increased as the birth cohort advanced, with more substantial increases in later birth cohorts for both men and women.

### Colorectal cancer

For colorectal cancer, a significant increase was found in both men and women during the study period, but the trend did not differ between men and women (APC, 20% for both men and women) (Table 1). The incidence trend for both groups significantly increased from 2001 to 2008, and then became approximately stable. The decrease in men was greater than the decrease in women (Figure 1). In men, the highest APC was found in the age group of 60 to 69 years, and in women, the highest APC was found in the age group of 50 to 59 years (APC, 24.2% for men; APC, 25.1% for women) (Table 2). The ASRs of colorectal cancer by birth cohort are plotted in Figure 2. The incidence increased in later birth cohorts for both men and women.

### Esophageal cancer

A significant increase in the incidence of esophageal cancer in men and women was observed from 2001 to 2010 (APC, 12% for men; APC, 11.2% for women) (Table 1). No difference in the trend of the incidence rate of esophageal cancer was found between men and women. A significantly increasing trend was found from 2001 to 2008, while it decreased from 2008 to 2010 (Figure 1). In both men and women, the highest increase in the APC was observed individuals 80 years of age or older (APC, 17.5% in men; APC, 15.3% in women) (Table 2). The ASIRs of esophageal cancer by birth cohort are plotted in Figure 2. The incidence increased in later birth cohorts in both men and women.

## DISCUSSION

This study documented the incidence of gastrointestinal cancers in Iran during a 10-year period. The cancers studied showed an increasing trend during the study period. Two reasons for this increase can be proposed: first, changes in lifestyle and food patterns to become more similar to those of Western countries, and second, improvements in the diagnosis and registration of cancer cases.

Evidence indicates that *Helicobacter pylori* is a cause of stomach cancer, and the decreasing trend of stomach cancer in developed countries has been found to be significantly related to the decrease of infections with this bacterium. The International Agency for Research on Cancer has reported that *H. pylori* is the main reason for stomach cancer [9]. The prevalence of this bacterium in developing countries, such as Iran, is 80%, whereas its prevalence in developed countries is 30% [10]. Therefore, the main reason for the increase in stomach cancer among Iranians is likely to be infections with this bacterium. The 5% decrease in the incidence trend of this cancer in men from 2008 to 2010 may have been related to the increase of antibiotic use to treat *H. pylori* infections. In most studies, it has been reported that salt and salty foods can increase the incidence of this cancer, while the consumption of fruits, fresh vegetables, and fish can have a protective effect [11-13]. Although a healthy diet including fruits and vegetables prevails in Iran, in comparison with developed countries, in recent decades it has been observed that dietary trends are moving toward Western foods, such as conserved and fast foods [14].

Smoking is a risk factor for gastrointestinal cancers. In a cohort study, it was reported that the risk of stomach cancer in smokers was twice that in non-smokers, and in the majority of studies, a significant relationship between smoking and cancer was found [15-17]. In general, the trend in stomach cancer incidence is decreasing simultaneously with the trend of smoking in developed countries, while these trends are concomitantly increasing in developing countries. Although we do not have exact information about smoking trends in the last decade in Iran, data about smoking in the 1990s in the country indicated that cigarette smoking prevalence decreased overall from 11.7 to 14.6% among individuals 15 to 69 years of age [18]. It seems that smoking has not had a major effect on stomach cancer incidence in Iran. Men gender is a risk factor for stomach cancer. In the current study, the incidence of stomach cancer in men was much higher than that in women. In some studies, this difference has been attributed to the protective role of woman hormones [19].

Many studies have shown an increase in stomach cancer in Iran. Haidari et al. [20] found that the overall incidence rate of stomach cancer increased from 2.8 per 100,000 in 2000 to 9.1 per 100,000 in 2005; moreover, Sadjadi et al. [21] in Ardabil province documented increase in the incidence of stomach cancer. Another study in Tehran and Shiraz indicated that the overall occurrence of stomach cancer slightly increased (0.08 for Tehran, and 0.03 for Shiraz) [22]. While Iran is facing an increase in the incidence of stomach cancer, other research has suggested a decrease in stomach cancer incidence in some countries, such as Cyprus, Jordan, Egypt, South America, and European locations such as Spain and the state of Amberia in Italy. The decline in the incidence of stomach cancer in the US and Western Europe may have resulted from *H. pylori* infection control and the impact of cancer control programs.

According to GLOBOCAN 2012, colorectal cancer in Iran is the fourth most prevalent cancer among men and the second most prevalent among women [23]. In a systematic review in Iran, the ASRs of colorectal cancer were 8.16 per 100,000 and 6.17 per 100,000 for men and women, respectively [24]. Najafi et al. [25] showed that the incidence of colorectal cancer in 1993 to 2007 increased by 14%, which is consistent with our results. Additionally, in a report from Fars province, the ASR for colon cancer among men was 1.61 per 100,000 from 1970 to 1980 and 4.20 per 100,000 from 1990 to 2000, with a significant annual increase of 0.13 per 100,000 (p<0.05) [26]. Yazdizadeh et al. [22] concluded that the incidence of colon cancer increased in Fars and Tehran provinces during the past 30 years. The magnitude of this increase, as measured by comparing the last five years to the first five years, was 82% for Tehran and 65% for Shiraz.

In some European studies, colorectal cancer showed a trend of increasing incidence from 1984 to 2007 [27,28]. Moreover, in some Asian countries, such as China, Japan, South Korea, and Singapore, a 2-fold to 4-fold increase in colorectal cancer has been reported during the past decades [29]. Similarly, the current study detected a considerable increase in cancer incidence among men and women during the study period.

Diet plays an important role in the development of colorectal cancer. Several cohort studies have revealed that consuming a considerable amount of fruits, vegetables, fish, and high-fiber foods can greatly decrease the incidence of this cancer [30,31]. One of the reasons for the increasing trend of colorectal cancer in Iran may be changes in dietary habits. Fast foods, which are typical of Western foods, are increasingly replacing Iranian traditional foods, which are mostly high in fiber [32]. Studies conducted in Iran have indicated that fat consumption increased greatly from 1990 to 2000, and that this increase was accompanied by an increase in the obesity rate [33,34]. Obesity, especially in the abdominal region, increases the risk of colorectal cancer by up to 40% in men and 16% in women [35]. Physical activity and maintaining an ideal weight can lead to a significant decrease in the incidence of colorectal cancer, as physically active people are 20 to 30% less affected by this cancer [36]. Only 31% of the mature population of Iran engages in regular physical activity [14]. Consequently, obesity and inadequate physical activity are other reasons for the increased incidence of colorectal cancer among Iranians. Previous studies in North America indicated that the risk of cancer for 15 to 20% of the population can be attributed to smoking habits [36]. The smoking prevalence in the mature population of Iran is approximately 14% [14]. In addition, colorectal cancer incidence increases with age. The risk of colorectal cancer increases slightly after 40 years of age, but after 50, it increases rapidly [11,37]. Such a pattern was observed in the current study; that is, the most positive trend of incidence was identified in men and women who were born in 1923 (age group of 80 to 84 years).

Iran is considered to be a country in the esophageal cancer belt, with a high incidence of this disease , and the highest incidence rates have been reported from northern provinces such as Mazandaran, Golestan, and Khorasan [38]. According to studies, Gonbad-e Kavus in Golestan province, with an incidence rate of 100 per 100,000 individuals, has one of the greatest cancer incidence rates in Iran and the world. In contrast, Kerman province, with an incidence rate of three per 100,000 individuals, is considered to have a low risk by worldwide standards [39].

Esophageal cancer is related to smoking habits and alcohol consumption. These factors, as well as the low consumption of fruits and vegetables, may account for 89% of the cases of this disease [40]. According to previous studies, the rate of esophageal cancer incidence in northeastern regions of Golestan province has decreased by half during the last 35 years. This considerable decrease took place after great improvements in the economic, social, and cultural status of the area [41]. The reasons for high rates of esophageal cancer incidence in Iran’s northeast have been analyzed in various studies during the past three decades, especially the last 10 years. The main reasons have been identified as drinking hot tea, not consuming enough fruits and vegetables, using drugs and their derivatives, drinking unhealthy water, and poverty. Moreover, genetic factors among the population of that area (Iran’s northeast) have been established as another reason for the high prevalence of this cancer [42-44].

Many studies in Iran have shown a reduced incidence of esophageal cancer. The majority of such studies were conducted before 2000. In contrast, our study identified an increased incidence during the study period. Part of this inconsistency may be due to differences in survey techniques and the size of the population studied. During the 1970s, the diagnosis of esophageal cancer was confirmed histologically in approximately 27% of cases [45], while in 2009 histological conformation was performed in 86% of cases [46]. It is likely, therefore, that the results obtained at that point reflected an overestimation of the true diagnosis of esophageal cancer. Improved diagnostic facilities, with greater accessibility of endoscopy for the diagnosis and the exact localization of upper gastrointestinal cancer, which plays a particularly important role in distinguishing cardia cancer from distal esophageal cancer, may be another reason for the decrease in esophageal cancer found in previous studies.

Part of the observed changes in the incidence of gastrointestinal cancer may be related to improved diagnostic techniques and more efficient registration. Until 2004, the cancer registry was only based on pathology reports. Subsequently, the population-based registration program in Iran expanded. According to a report of the Iranian Ministry of Health, the coverage of the cancer registry in Iran has increased from 18% in 1999 to 81% in 2005 [46]. A study in Italy showed that a high proportion of the increase in the incidence of stomach cancer was related to improvements in diagnostic techniques and the cancer registry system [47]. Additionally, previous studies in Iran have explicitly discussed the improvement in the cancer registry in Iran [48,49].

Gastrointestinal cancers showed a trend of increasing incidence in both genders from 2000 to 2010. The observed trends are associated with changes in lifestyle, socioeconomic conditions, and increased life expectancy. Therefore, it is essential to place a primary focus on prevention methods in order to reduce the incidence of cancer.

## Figures and Tables

**Table 1.** CRs and ASRs for gastrointestinal cancers in men and women in Iran, 2001-2010

Cancer site	Men							Women						
	2001			2010				2001			2010			
	No. of cases	CR	ASR	No. of cases	CR	ASR	APC	No. of cases	CR	ASR	No. of cases	CR	ASR	APC
Stomach	1,105	3.3	4.18	5,192	13.2	17.06	16.7^*^	484	1.5	2.41	2,238	6.5	8.85	16.2^*^
Colorectal	379	1.3	2.12	3,443	8.8	11.28	20.0^*^	344	1.2	2.00	2,641	7.7	10.33	20.0^*^
Esophageal	858	2.5	3.25	1,693	4.3	5.57	12.0^*^	405	1.3	2.10	1,378	4.0	5.62	11.2^*^

CR, crude rates (per 100,000); ASR, age-standardized incidence rates (per 100,000); APC, annual percent change of ASRs.

* p<0.05.

**Table 2.** Trends in the ASRs for gastrointestinal cancers in Iranian men and women according to a joinpoint analysis by age group (2001-2010)

Cancer site, age	APC (95% Cl) 2001-2010	Trend 1	APC (95% CI)	Trend 2	APC (95% CI)	Trend 3	APC (95% CI)
Men							
Stomach							
40-49	12.4 (0.6, 25.7)	2001-2003	54.2 (-21.8, 204.0)	2003-2008	11.9 (-9.7, 38.7)	2008-2010	-17 (-57.9, 63.7)
50-59	9.8 (6.3, 13.3)	2001-2003	41.4 (16.4, 71.7)	2003-2008	10.2 (3.6, 17.2)	2008-2010	-15.6 (-30.5, 2.5)
60-69	12.3 (6.5, 18.4)^*^	2001-2003	41.5 (6.5, 88.0)^*^	2003-2010	5.1 (1.2, 9.2)	N/A	N/A
70-79	16.6 (12.4, 21.0)	2001-2003	63.4 (30.6, 104.4)	2003-2008	9.6 (2.1, 17.6)	2008-2010	-2.9 (-22.3, 21.5)
80+	23.7 (14.3, 33.9)	2001-2004	63.6 (26.3, 112.0)	2004-2010	7.6 (-1.5, 17.4)	N/A	N/A
All	16.7 (14.6, 18.8)^*^	2001-2003	70.4 (52.6, 90.3)^*^	2003-2008	8.9 (5.1, 12.8)^*^	2008-2010	-5.1 (-15.0, 6.0)
Colorectal							
40-49	13.0 (10.4, 15.6)^*^	2001-2003	46.7 (27.6, 68.6)^*^	2003-2008	15.1 (10.1, 20.3)^*^	2008-2010	-16.9 (-27.7, -4.5)^*^
50-59	17.2 (11.8, 22.8)^*^	2001-2003	52.9 (15.0, 103.3)^*^	2003-2008	17.6 (7.5, 28.7)^*^	2008-2010	-11.0 (-33.1, 18.3)
60-69	24.2 (16.1, 32.9)^*^	2001-2003	62.3 (13.1, 133.1)^*^	2003-2010	15.1 (9.7, 20.8)^*^	N/A	N/A
70-79	21.0 (15.4, 26.9)^*^	2001-2004	43.1 (22.7, 66.9)^*^	2004-2010	11.3 (5.6, 17.2)^*^	N/A	N/A
80+	21.2 (11.9, 31.3)^*^	2001-2007	35.9 (24.4.8, 48.4)^*^	2007-2010	-3.6 (-25.7, 25.2)	N/A	N/A
All	20.0 (15.6, 24.6)^*^	2001-2003	63.3 (30.0, 105.1)^*^	2003-2008	15.9 (7.8, 24.5)^*^	2008-2010	-3.8 (-23.4, -0.7)
Esophageal							
40-49	9.2 (-6.0, 26.8)	2001-2004	42.8 (-10.0, 126.5)	2004-2008	6.2 (-33.1, 38.4)	2008-2010	-22.9(-69.4, 94.1)
50-59	12.5 (6.5, 18.8)^*^	2001-2003	64.3 (17.6, 129.6)^*^	2003-2008	10.8 (-0.3, 23.2)	2008-2010	-20 (-42.7.2, 11.7)
60-69	14.3 (6.2, 23.1)^*^	2001-2003	57.7 (6.4, 133.7)^*^	2003-2010	4.3 (-1.0, 9.9)	N/A	N/A
70-79	13.4 (5.2, 22.2)^*^	2001-2005	31.7 (11.6, 55.5)^*^	2005-2010	0.6 (-10.6, 13.1)	N/A	N/A
80+	17.5 (6.4, 29.8)^*^	2001-2003	77.1 (-3.3, 224.4)	2003-2008	15.5 (-4.6, 39.9)	2008-2010	-18.6 (-55.6, 49.0)
All	12.0 (6.6, 17.7)^*^	2001-2003	54.6 (14.1, 109.4)^*^	2003-2008	8.9 (-1.1, 19.9)	2008-2010	-12.8 (-35.7, 18.1)
Women							
Stomach							
40-49	12.1 (3.4, 21.6)	2001-2004	38.3 (13.0, 69.3)	2004-2008	12.3 (-6.3, 34.6)	2008-2010	-18.5 (-57.3, 55.3)
50-59	14.6 (8.5, 21.0)	2001-2004	35.8 (13.8, 62.1)	2004-2010	5.3 (-0.9, 11.7)	N/A	N/A
60-69	13.1 (5.9, 20.8)^*^	2001-2004	35.6 (9.5, 68.0)^*^	2004-2010	3.3 (-3.9, 11.0)	N/A	N/A
70-79	16.1 (8.8, 23.9)	2001-2003	40.1 (-1.2, 98.7)	2003-2010	10.0 (5.0, 15.3)	N/A	N/A
80+	18.6 (13.9, 23.5)	N/A	N/A	N/A	N/A	N/A	N/A
All	16.2 (7.7, 25.4)^*^	2001-2003	53.3 (2.1, 130.1)^*^	2003-2010	7.4 (1.7, 13.4)^*^	N/A	N/A
Colorectal							
40-49	18.6 (8.3, 29.8)^*^	N/A	N/A	N/A	N/A	N/A	N/A
50-59	25.1 (13.3, 38.2)^*^	2001-2003	69.3 (-0.3, 187.5)	2003-2010	14.8 (6.9, 23.2)^*^	N/A	N/A
60-69	19.5 (13.3, 26.1)^*^	2001-2004	52.8 (28.5, 81.8)^*^	2004-2010	5.7 (-0.3, 12.1)	N/A	N/A
70-79	20.2 (15.5, 25.1)^*^	2001-2004	48.3 (30.1,69.1)^*^	2004-2010	8.2 (3.6, 13.1)^*^	N/A	N/A
80+	23.0 (10.9, 36.6)^*^	2001-2004	60.4 (14.2, 125.3)^*^	2004-2010	7.8 (-3.9, 20.9)	-	-
All	20.0 (18.8, 21.3)^*^	2001-2003	66.8 (56.4, 78.0)^*^	2003-2008	13.8 (11.5, 16.1)^*^	2008-2010	-1.3 (-7.5, 5.3)
Esophageal							
40-49	6.5 (-1.8, 15.4)	2001-2003	62.7 (-0.4, 165.9)	2003-2008	1.9 (-1.7, 19.0)	2008-2010	-22.3 (-52.4, 27.0)
50-59	14.0 (1.5, 28.2)^*^	2001-2003	52.8 (-18.2, 185.6)	2003-2010	4.9 (-3.5, 14.0)	N/A	N/A
60-69	13.9 (5.6, 22.9)^*^	2001-2003	70.8 (14.0, 155.8)^*^	2003-2010	1.5 (-3.9, 7.1)	N/A	N/A
70-79	12.6 (3.7, 22.4)^*^	2001-2003	52.3 (-8.1, 152.5)	2003-2008	9.7 (-6.5, 28.7)	2008-2010	-11.1 (-46.4, 47.4)
80+	15.3 (4.9, 26.8)^*^	2001-2003	28.7 (20.2, 37.7)^*^	2003-2010	-21.5(-52.7, 30.4)	N/A	N/A
All	11.2 (6.2, 16.5)^*^	2001-2003	54.5 (16.7, 104.7)^*^	2003-2008	7.4 (-1.7, 17.4)	2008-2010	-12.7 (-34.1, 15.6)

ASR, age-standardized incidence rate; APC, annual percentage change of ASRs; CI, confidence interval; N/A, not applicable.

* p<0.05.
